# The unimodal distribution of sub–threshold, ongoing activity in cortical networks

**DOI:** 10.3389/fncir.2013.00116

**Published:** 2013-07-11

**Authors:** Anat Yaron-Jakoubovitch, Christof Koch, Idan Segev, Yosef Yarom

**Affiliations:** ^1^Department of Neurobiology, The Hebrew UniversityJerusalem, Israel; ^2^The Interdisciplinary Centre for Neural Computation, The Edmond and Lily Safra Center for Brain Sciences The Hebrew UniversityJerusalem, Israel; ^3^Allen Institute for Brain ScienceSeattle, WA, USA

**Keywords:** *in vivo*, ongoing activity, up and down states, subthreshold, oscillations

## Abstract

The characterization of the subthreshold, ongoing activity in cortical neurons has been the focus of numerous studies. This activity, described as spontaneous slow waves in membrane potential, has been observed in a span of species in diverse cortical and subcortical areas. We here characterized membrane potential fluctuations in motor and the frontal association cortices cortical neurons of ketamine–xylazine anesthetized rats. We recorded from 95 neurons from a range of cortical depths to unravel the network and cellular mechanisms that shape the subthreshold ongoing spontaneous activity of these neurons. We define a unitary event that generates the subthreshold ongoing activity: giant synaptic potentials (GSPs). These events have a duration of 87 ± 50 ms and an amplitude of 19 ± 6.4 mV. They occur at a frequency of 3.7 ± 0.8 Hz and involve an increase in conductance change of 22 ± 21%. GSPs are mainly due to excitatory activity that occurs throughout all cortical layers, unaffected by the intrinsic properties of the cells. Indeed, blocking the GABA_A_ receptors, a procedure that had a profound effect on cortical activity, did not alter these unitary events. We propose that this unitary event is composed of individual, excitatory synaptic potentials that appear at different levels of synchrony and that the level of synchrony determines the shape of the subthreshold activity.

## INTRODUCTION

The characterization of spontaneous slow waves in the membrane potential of cortical neurons has been the focus of numerous studies. These waves have been observed in a range of species in diverse cortical (Destexhe et al., 2003; Haider et al., 2006, 2007; Waters and Helmchen, 2006; Nita et al., 2007; Rudolph et al., 2007; Okun et al., 2010) and subcortical (Destexhe et al., 2007; Ji and Wilson, 2007) areas. The basic characterizations of the time course of these slow waves vary. At one end of the spectrum are the oscillations manifested in the form of up and down states (Steriade et al., 1993; Lampl et al., 1999; Petersen et al., 2003; Sachdev et al., 2004; Crochet et al., 2005; Leger et al., 2005; Hahn et al., 2006, 2007; Haider et al., 2006, 2007; Hasenstaub et al., 2007; Poulet and Petersen, 2008). At the other end are those manifested as bumps (Las et al., 2005; DeWeese and Zador, 2006; Rudolph et al., 2007; Okun and Lampl, 2008) or transient depolarizations (Azouz and Gray, 2008). Furthermore, Anderson et al. (2000) reports oscillations with a duration longer than that of "bumps" yet shorter than that of up and down states. This suggests that the slow spontaneous oscillations exist in a spectrum of amplitudes and durations. Another controversial issue concerns the existence and extent of the change in conductance that are involved in the slow oscillation. Some reports have found no change or a small decrease in conductance (Wilson and Groves, 1981; Wilson and Kawaguchi, 1996; Waters and Helmchen, 2006) while others show a two to fivefold increase in conductance (relative to the input conductance) at the peak of the oscillation (Pare et al., 1998; Steriade et al., 2001; Destexhe et al., 2003; Petersen et al., 2003; Crochet et al., 2005; Leger et al., 2005; Rudolph et al., 2007).

Although it is commonly accepted that these up and down alternations are a network phenomenon, the source of these oscillations remains unexplored. It has been suggested that the slow oscillation in the prefrontal cortex are initiated in layer 5, propagating then onto layers 2/3 and 6 (Sanchez-Vives and McCormick, 2000). In neocortical areas 5 and 7, activity has been shown to commence in layer 5 as well (Chauvette et al., 2010). Since the spontaneous activity remains after a cortical area is deafferented, it appears that the slow cortical oscillations can be generated entirely by local cortical synaptic connections (Timofeev et al., 2000). Furthermore, slow oscillations in cortical slices can emerge spontaneously (Sanchez-Vives and McCormick, 2000), or can be evoked by thalamic stimulation (MacLean et al., 2005).

The functional significance of this activity is unclear. Destexhe et al. (2007) suggest that up states may be fragments of wakefulness during sleep. However, the up and down states during slow wave sleep do not lead to conscious experience. Alternatively, it has been suggested that the up and down states are a replay of awake experience during sleep, involved in memory consolidation (Marshall et al., 2006; Ji and Wilson, 2007).

In view of these multiple, sometimes contradictory, observations we conducted a thorough study on a large population of neurons that span through all cortical layers as well as two different cortical areas in an attempt to understand the underlying biophysical mechanisms of the subthreshold rhythmic activity of cortical tissue as well as the source for the contradictory observations. To that end, we recorded from 95 cortical neurons of ketamine–xylazine anesthetized rats. Our data shows that the periodic depolarizing events, that we defined as the unitary event that composes the subthreshold ongoing activity [giant synaptic potentials (GSP)], are mainly due to excitatory activity that occurs throughout the cortical layers, unaffected by the intrinsic properties of the cells.

We postulated that up and down states, bumps, transient depolarizations and our GSPs arise from similar network mechanisms, as suggested by DeWeese and Zador (2006). Different network synchronization levels may give rise to different manifestations of this oscillation.

## MATERIALS AND METHODS

All procedures used in the study adhere to guidelines approved by the Hebrew University of Jerusalem Animal Care Committee and comply with NIH guidelines.

### *IN VIVO* RECORDING

#### Animal surgery

Experiments were conducted on Sprague-Dawley rats weighing 20–75 g, in the age range of postnatal day 16–28. Animals were anesthetized with an initial dose of ketamine–xylazine (10:1 mg/kg) mixture. The depth of anesthesia was sufficient to eliminate pinch withdrawal and vibrissae movements. Anesthesia was maintained throughout experiments by supplements of ketamine (100 mg/kg) administered as necessary (typically every 0.5–1 h). The body temperature was kept at 37°C using a heating blanket and rectal thermometer. A craniotomy (2 by 2 mm) was opened over the motor and the frontal association cortices cortex (stereotactic coordinates: anterior 3–5 mm from bregma, 0.5–2.5 mm from the midline (Watson, 1998) and the dura was removed.

#### Electrophysiology

Whole cell patch clamp recordings were obtained using a “blind” technique. Recording pipettes (6–8 MΩ) were filled with an intracellular solution containing the following: 140 mM K-gluconate, 4 mM NaCl, 0.5 mM CaCl_2_, 5 mM EGTA, 3 mM MgATP, 10 mM HEPES, pH 7.2. After touching the brain with the recording pipette, the craniotomy was covered with agar (3–3.5%) in artificial cerebrospinal fluid (ACSF) containing: 125 mM NaCl, 2.5 mM KCl, 25 mM NaHCO_3__,_ 1.25 mM NaH_2_PO_4_, 1 mM MgCl_2_, 25 mM Glucose, 2 mM CaCl_2_.****Positive pressure (>200 mbar) was applied to the pipette as it was inserted into the cortex. The positive pressure was reduced to 25–30 mbar when the tip was at approximately the upper limit of L2/3. The pipette was then advanced in 2–3 μm steps. Current pulses were applied to the pipette (100 pA, 10 Hz), and the voltage response was monitored. Positive pressure was relieved when the resistance of the electrode abruptly increased, indicating that the tip of the pipette may have touched a cell membrane. Negative pressure was then applied in order to create a Giga-Ohm seal. The membrane was then ruptured using strong suction. The membrane was ruptured after achieving a 5 Giga-Ohm seal. The electrode’s series resistance was typically between 60 and 80 Mega-Ohm after rupturing the membrane.****

#### Cell labelling

For intracellular labeling, biocytin (1%; Sigma) was included in the intracellular solution. 150 ms positive current pulses of up to 1 nA in amplitude and 3 Hz frequency for at least 20 min were used to deliver the biocytin into the cell.

After the experiment the animal was perfused through the heart with phosphate buffer solution (PBS) followed by fixative solution containing 4% formaldehyde diluted in PBS. The brain was isolated and incubated over night in the fixative solution. After washing out the fixative, 80 μm thick sagittal sections were cut from a tissue block containing the area where recordings were performed. Endogenous peroxidases were quenched by a 5-min incubation in 1% H_2_O_2_. Tissue was reacted (5–6 h) in avidin–biotin complex (ABC Elite kit; Vector Laboratories, Burlingame, CA, USA) after which biocytin was demonstrated by 3,3′-diaminobenzidine tetrahydrochloride (DAB; Sigma) histochemistry. Subsequently, the slices were mounted on objective glass under coverslip and digitally imaged using a Leica TCS SP5 microscope.

**FIGURE 1:**
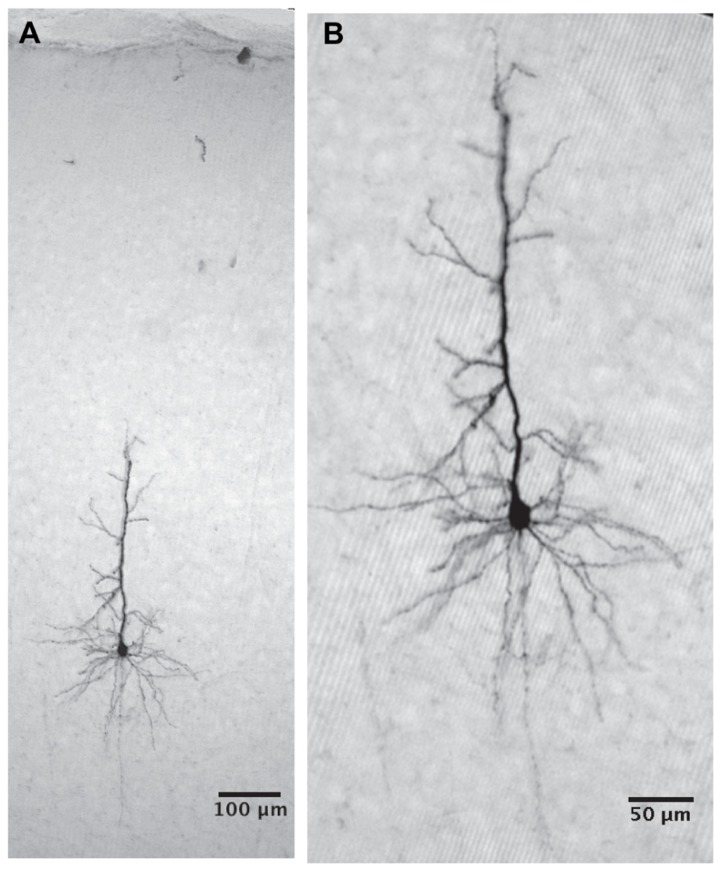
**The cell was intracellularly injected with biocytin and developed with DAB.** **(A)** The cell body is located 921 micron below the cortical surface and the apical dendrite reaches the cortical surface (the tip of the apical dendrite appears in the next histological section). **(B)** A higher magnification reveals a typical layer 5 pyramidal cell. The deep location of the pyramidal cell strongly supports our claim that we measure from all cortical layers.

**FIGURE 2:**
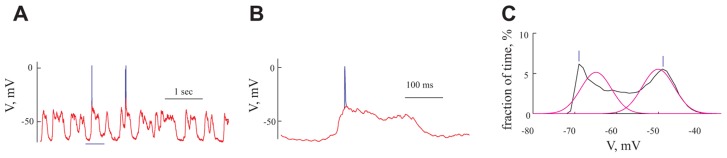
**Analysis of intracellular voltage traces (A) Voltage trace recorded from a cell at resting potential**. Spikes are shown in blue, overlapped by the voltage trace after removal of the spikes (red). **(B)** Expansion of 500 ms from **(A)** (bounded by the blue horizontal line in **A**).**(C)** The distribution of membrane voltage (black line) calculated from the voltage trace in **(A)** after removal of spikes. The voltage distribution was fitted by two Gaussians (magenta line). Vertical lines bars denote the visually identified peaks that were used to construct the voltage-current relationship for the “histogram peaks methods” (see Materials and Methods)

As shown in **Figure 1A** cells were labeled as deep as 921 microns from the surface. Higher gain examination (**Figure 1B**) reveals that in this case it was a typical layer 5 pyramidal cell. Thus our population of neurons indeed covers all cortical layers. The labeled cell is a typical deep layer 5 pyramidal cell (**Figure 2**) where the apical dendrite, whose tip appears in the next histological section, extends all the way to the pia matter.

### DATA ACQUISITION

Recordings were performed using a Multiclamp 700B amplifier (Molecular Devices, Union City, CA, USA) and were sampled by a National Instruments board (PCI-MIO-16XE) at a rate of 10 kHz. A custom-built interface written in the LabVIEW environment (V7, National Instruments, Austin, TX, USA) was used for data acquisition. We recorded the voltage from each neuron for 1–2 min at resting potential. Different levels of DC currents of up to ±600 pA were then injected into the cell, shifting the membrane potential from -80 to -40 mV. Recordings were performed for additional 1–2 min at each membrane potential level.

### DATA ANALYSIS

All data were analyzed offline using MATLAB (The MathWorks, Natick, MA, USA). Spikes were removed from the data (from 1 ms before to 10 ms after the spike) and replaced by a line connecting the endpoints of the removed segment (Figures 2A,B).

#### Criteria for choosing cells for analysis

We recorded from more than 100 cells. We chose for analysis only cells with a resting potential hyperpolarized to -50 mV, overshooting action potentials (APs) and adequate bridge balance.

#### Offline bridge correction

Cells which did not have a stable resting potential throughout the recording session were omitted from the data set. The bridge was balanced at the beginning of the recording at each membrane potential and was re-examined at the end of the recording session. However, despite the careful monitoring of changes in access resistance during the recording, minor changes can occur. As our analysis was prone to errors caused by changes in access resistance or incorrect bridge settings, we corrected the bridge balance offline using method of Frank and Fuortes (1956). The peak of the AP has a maximal conductance and therefore its value is independent of the initial condition potential. Only cells for which an adequate bridge balance was achieved were selected for further analysis. After all these procedures we ended up with 95 cells.

#### Analysis of giant synaptic potentials *in vivo*

Our data shows periodic depolarizing events, GSPs. Three different methods were used to differentiate between resting potential and GSP peak.

**FIGURE 3:**
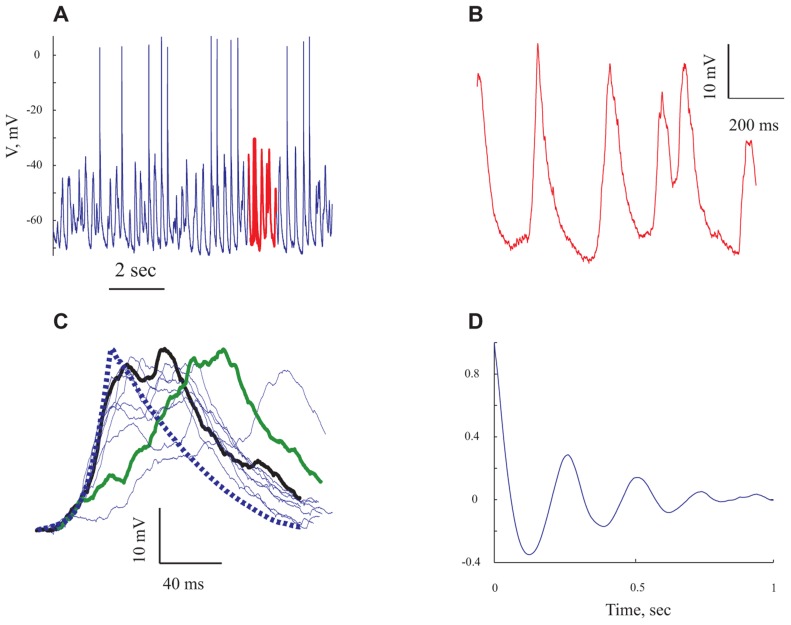
**The rhythmic subthreshold spontaneous activity is composed of individual gigantic synaptic potentials that appear at different degrees of synchrony.** **(A)** A 10 s long voltage trace recorded at resting potential. **(B)** Expansion of the section marked in red in **(A)**. **(C)** The giant synaptic potential (GSP) time course. Thin lines denote single GPSs, thick blue line denotes an average of 247 GSPs. Thick green line denotes an example of a "staircase"-like GSP. The thick black line is an example of a GSP with a similar rise time to that of the average GPS. **(D)** An autocorrelogram of the voltage trace shown in **(A)** All panels refer to data from the same cell.

1. Histogram peak: We calculated voltage distributions for each membrane potential level (**Figure 2C**). Only cells showing a clear bimodal voltage distribution (*n* = 46) resulting from the GSPs (**Figure 2C** black line) were used for this analysis. At each membrane potential level we visually defined the peaks of the histogram (bars in **Figure 2C**, for the peak of GSP and resting potential, respectively).

2. Gaussian fitting: As shown in **Figure 2C** (magenta line), two Gaussians were fitted to the voltage histograms described above using hard maximum likelihood iterative clustering; data were clustered into two clusters and each was fitted with a Gaussian. The match between the data and the Gaussians was assessed using a maximum likelihood approach. This was carried out iteratively with a stopping criterion of 0.01 mV. The peaks of the Gaussians were considered as the membrane potentials for the resting potential (left peak) and GSPs (right peak). Using this method, all cells were analyzed (*n* = 95).

3. Averaging GSP events: For each voltage trace a threshold was defined as the mean membrane potential + standard deviation. When the membrane potential crossed the threshold, a GSP event was determined. Each threshold crossing was parsed from 100 ms before to 250 ms after the peak of the GSP. All such events were averaged and aligned on the onset of the GSP. The membrane potential prior to the GSP was considered as the resting potential and the peak voltage was considered as the GSP potential (**Figure 3C**).

#### Amplitude of GSPs

The amplitude of GSPs was calculated as the voltage difference between rest (minimal voltage before GSP onset) and the peak of the GSP. Calculating the amplitude using the other two methods (Gaussian fitting and histogram peaks) was carried out by subtracting the value of the hyperpolarized peak from that of the depolarized peak. To partially compensate for the effect of different resting potentials between different cells, we normalized the amplitude according to the following ratio:

Normalized amplitude = amplitude * (-89/Vrest), where -89 mV is the lowest resting potential in our population of cells and Vrest is the individual resting potential.

#### Rise and decay time of the GSPs

The rise time of the GSP was calculated as the time it takes the voltage to shift from 10 to 90% of the amplitude of the GSP.

#### Duration of GSPs

The period of time of the GSP was measured as its width at half of the amplitude.

#### Frequency of GSPs

The frequency of GSP was defined as the number of GSP events divided by the time of recording (1–2 min).

#### Input resistance measurement

**FIGURE 4:**
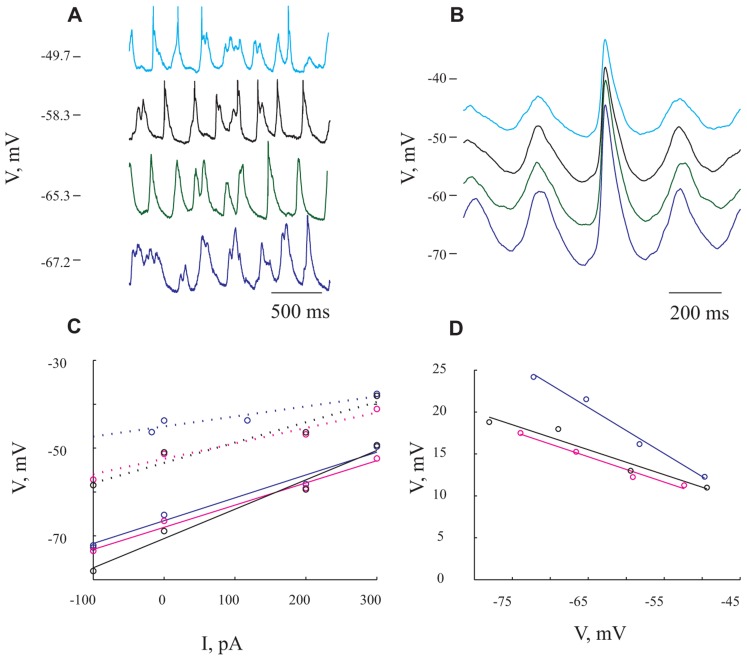
**Giant synaptic potentials are associated with increased conductance.** **(A)** Voltage traces at 4 different membrane potentials resulting from DC current injection of -100, 0, 200, and 300 pA in blue, green, black, and cyan, respectively. Spikes have been truncated for illustration purposes. **(B)** Averaged of several hundreds of GSPs from the same membrane potentials shown in **(A)**. **(C)** I/V curves for resting potential (solid lines) and peak of the GSP (dashed lines). Blue lines denote I/V curves calculated from the average GSP method. The *R*_in_ values that were obtained using this method were 52 and 23 MΩ for resting potential and GSP peak, respectively. In black and magenta are I/V curves calculated from the histogram peaks (resulting in *R*_in_ of 79 and 49 MΩ for resting potential and GSP peak, respectively) and Gaussian fitting (resulting in *R*_in_ of 50 and 35 MΩ for resting potential and GSP peak) methods, respectively (see Materials and Methods). **(D)** GSP amplitude as a function of membrane potential. Note the steep decrease in GSP amplitude with depolarization (same colors as in **C**). The expected reversal potential of the GSPs is calculated by extrapolation of the curves to intersect with the abscissa. All panels refer to data from the same cell.

We calculated IV curves for the voltage at rest and for the voltage at the peak of the GSP (see below) using the three methods to define the resting and the peak voltage and the DC level that was injected (**Figure 4**). We then calculated the input resistance (*R*_in_) from the IV curves by extracting the slope from a line fitted to the curve at its linear regime (**Figure 4**). The relative change in resistance was calculated as [*R*_in_(rest)-*R*_in_(GSP peak)]/*R*_in_(rest).

### PHARMACOLOGY

GABA_A_ mediated inhibitory synaptic activity was blocked when needed using gabazine (2.5 μM, at a total volume of 0.05 mL, Sigma-Aldrich, in ACSF solution). In these experiments we skipped the agar application procedure in order to expose the cortical area to the direct gabazine application.

## RESULTS

### SPONTANEOUS RHYTHMIC GIANTS

**FIGURE 5:**
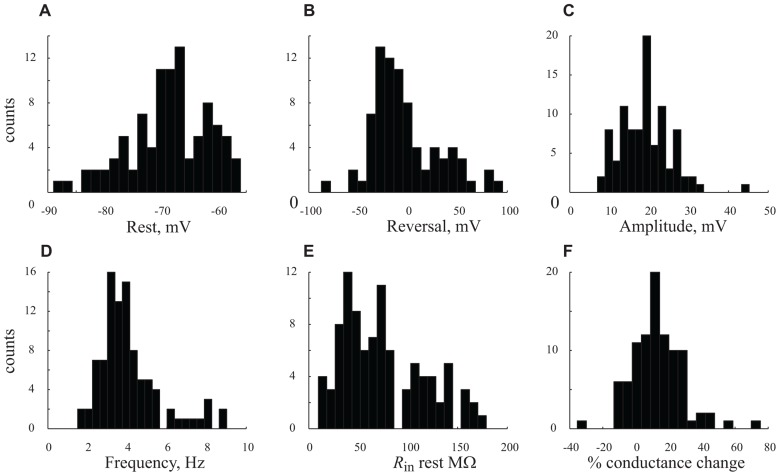
**Distribution the cells’ characteristics.** **(A–F)**. Histograms denote results obtained from all *n* = 56 cells under control conditions **(A)** resting potential. **(B)** GSP reversal potential. **(C)** GSP amplitude. **(D)** GPS frequency. **(E)** *R*_in_ at resting potential. **(F)** Percentage (%) of conductance change between resting potential and peak of GSP. Note the unimodal distributions of all the parameters.

Spontaneous activity was recorded from 95 pyramidal neurons of the prefrontal cortex under ketamine–xylazine anesthesia. The cells had an average resting potential of -68.5 ± 7.1 mV (**Figure 5A** and **Table 1**) and all expressed rhythmic, large voltage fluctuations, resembling a GSP, (**Figures 2A, B, 3A, B, and 4A,B**). An expanded time scale (**Figures 2B and 3B**) reveals that although the GSPs are variable in size and shape, they all have a fast rise time and a slower decay time. A careful examination of the rise time shows that occasionally it resembles a staircase like structure (**Figure 3C** thick green line), possibly denoting temporal summation of individual synaptic potentials. On other occasions it has a smooth rise time (**Figure 3C** thick black line).

**Table 1:** A summary of the cells’ characteristics using different intracellular solutions.

	Resting (mV)	Reversal (mV)	Amplitude (mV)	*R*_ in _ change (%)	GSP Freq (Hz)	Spike rate (Hz)	*R*_ in _ rest (Mø)	*R*_ in _ GSP (Mø)	Rise (ms)	Duration (ms)	Depth (μm)	Age (days)
Control	-68.5± 7.1	-8 ± 23.8	19 ± 6.4	22 ± 21	3.7 ± 0.8	0.52 ± 0.47	74.4 ± 43	59 ± 39	37 ± 18	77 ± 28	538 ± 184	21.4 ± 3.9
	*n* = 95	*n* = 69	*n* = 95	*n* = 95	*n* = 84	*n* = 85	*n* = 95	*n* = 95	*n* = 83	*n* = 95	*n* = 95	*n* = 95
Cl	-48.9 ± 23.5	-14.4 ± 67.7	16.2 ± 6.7	5 ± 16	3.6 ± 1	0.77 ± 0.85	62.1 ± 2.4	57.6 ± 27.5	54 ± 14	88 ± 30	499 ± 130	27.6 ± 3
	*n* = 9	*n* = 9	*n* = 8	*n* = 9	*n* = 9	*n* = 9	*n* = 9	*n* = 9	*n* = 9	*n* = 9	*n* = 9	*n* = 9

In order to quantify the characteristics of the GSPs in this cell, we averaged 247 GSPs (dashed thick blue line in **Figure 3C**). Indeed, the average GSP has a fast rise time (24 ms) and a rather long duration (69 ms), at half the amplitude. The average rise time of GSPs at resting potential for all recorded cells was 36 ± 43 ms (37 ± 18 after omitting 12 cells with extreme values; **Table 1**). The average duration of the GSP across all recorded cells was 87 ± 50 ms. Recalculating the average GSP duration after omitting 9/95 neurons with exceptionally long durations did not change significantly the average duration (77 ms), but significantly reduced the standard deviation (28 ms, **Table 1**). The average amplitude of the GSPs of the cell in **Figure 3** was 21.3 mV; the average amplitude of GSPs at resting potential in all of the recorded cells was 19 ± 6.4 mV (**Figure 5C**, **Table 1**).

Action potentials, were elicited exclusively from the GSPs (**Figures 2A and 3A**) at a frequency of 0.83 ± 1.1 Hz, in agreement with Chauvette et al. (2010) but in contrast with Sakata and Harris (2009) and Hasenstaub et al. (2007). Omitting 10 cells with exceptionally high firing rates resulted in an average firing frequency of 0.52 ± 0.47 Hz (**Table 1**). **Figure 3D** shows an autocorrelogram of the voltage trace in **Figure 3A**, confirming the rhythmic appearance of the GSPs. The frequency of the GSPs in this cell was 4.1 Hz. The average frequency of GSPs in all recorded cells was 4.1 ± 1.5 Hz (**Figure 5D**). Omitting 11/95 cells with extreme frequencies yielded a frequency of 3.7 ± 0.8 Hz (**Table 1**).

### MEMBRANE CONDUCTANCE CHANGE DURING THE GSPs

To estimate the extent of conductance change during GSPs we tested their voltage dependence by injecting different DC currents into the cells (up to ±600 pA). In the example shown in **Figure 4**, the current injection shifted the membrane potential from -72 to -50 mV. Two points are immediately evident. First, there is an increase in firing rate (from an average of 1.4 Hz at the hyperpolarized membrane potential to 3.05 Hz at the depolarized membrane potential), without a prominent change in the frequency of the GSPs (from 4.2 Hz at the most hyperpolarized membrane potential to 4.6 Hz at the most depolarized membrane potential). Second, there was a substantial decrease in the average GSPs’ amplitude (compare cyan and blue traces in **Figure 4B**).

**Figure 4A** depicts the spontaneous activity measured at four different levels of membrane potentials. The extent of the decrease in amplitude was assessed using three different approaches (see Materials and Methods). First, averaging the GSPs revealed an almost twofold decrease in amplitude over 20 mV shift in membrane potential (compare blue and cyan lines in **Figure 4B**). Indeed, plotting the resting membrane potential (**Figure 4C**, continuous lines) and the membrane potential at the peak of GSP (**Figure 4C** dotted lines) as a function of the injected current revealed a linear relation (**Figure 4C**, blue lines). The difference between the two blue lines, which quantifies the decrease in amplitude, is demonstrated in **Figure 4D** in blue. The input resistance (*R*_in_) calculated by measuring the slope of a fitted linear curve to each of the lines was 52 and 23 MΩ for the resting and peak potentials, respectively. In the second approach we used the “Gaussian fitting procedure” (see Materials and Methods), yielding the magenta lines in **Figure 4C**, resulting in estimated *R*_in_ values of 50 and 35 MΩ for resting and peak potentials of the GSP, respectively. Third, we used the “histogram peak approach” (see Materials and Methods) that yielded the black lines in **Figure 4C** with estimated *R*_in_ values of 79 and 49 MΩ for resting and peak potentials of GSP, respectively.

All three methods show that GSPs in this cell are associated with a decrease in *R*_in_ at the peak of the GSP compared to the resting state. Using the average GSP method, the resting *R*_in_ for all recorded cells before the onset of the GSP was 74.4 ± 43 MΩ, significantly higher than 59 ± 39 MΩ at the peak of the GSP (*p* < 0.05, **Figures 5E, F**). The mean *R*_in_ change during the GSP (see Materials and Methods) of all recorded neurons was 22 ± 21%, ranging between -35 and 76% (**Table 1**). *R*_in_ values of 87 ± 44 and 70 ± 34 and 74.4 ± 41 and 65 ± 40 MΩ yielding *R*_in_ changes of 18 ± 19 and 13 ± 16% were obtained using the histogram peaks and Gaussian fitting methods, respectively. These results clearly demonstrate that the GSP is associated with an increased membrane conductance, further supporting the idea that GSPs are composed of synaptic processes. Seventy seven out of the 95 recorded cells showed an increase in membrane conductance at the peak of the GSP, 28 ± 18%, while only 16 cells showed a decrease in membrane conductance at the peak of the GSP of 9 ± 8%. The latter was calculated using the average GSP; the other two methods showed similar results.

### IONIC SPECIFICITY UNDERLYING THE GSP CONDUCTANCE CHANGE

To gain insight into the specificity of the conductance change associated with the GSP, we estimated the reversal potential of the GSP process. **Figure 4D** depicts the relationship between the GSP amplitude and the membrane potential, calculated by three different methods. In all three methods the linear relationship is evident and extrapolation to zero of the amplitude should yield the GSPs’ reversal potential. Using the averaged GSP, histogram peaks and Gaussian fitting methods we calculated reversal potentials of -27.6, -12.9, and -16.4 mV, respectively. We calculated the reversal potential for all of the recorded cells. Cells for which a reversal potential smaller than -100 mV or larger than 100 mV was calculated were considered as non-reversing. Using the averaged GSP method, the mean reversal potential for all recorded neurons was -3.3 ± 34.2 mV (*n* = 79, **Figure 5B**). Recalculating the average reversal potential after omitting 10/79 neurons with exceptionally high/low reversal potentials, indicating very low conductance changes, did not change significantly the reversal potential (-8 mV), but significantly reduced the standard deviation (±23.8 mV; **Table 1**). Calculating the reversal potential using the two other methods yielded reversal potentials of -13 ± 16 and -5.4 ± 22.3 mV for the histogram peaks and Gaussian fitting methods, respectively (when all cells are included).

In order to rule out a distortion of the reversal potential by membrane non-linearities (Waters and Helmchen, 2006), we recalculated the reversal potential only for cells which showed a linear behavior for an extended part of the IV curve (at least 30 mV). The 19 cells that fulfilled these conditions yielded an average reversal potential of -2 ± 17 mV, using the average GSP method.

Our relatively high reversal potential which was measured at the peak of the GSP may reflect a late contribution of the inhibitory synapses (Wilent and Contreras, 2005; Okun and Lampl, 2008). We therefore calculated the reversal potential at different times (20 and 40 ms) after the GSP peak. These calculations yielded reversal potentials which were not different from the reversal calculated at the peak of GSP (-2 ± 37 and -11 ± 32 mV for 20 and 40 ms after GSP peak, respectively, *p* > 0.05, Wilcoxon signed rank test).

The depolarized reversal potential indicates that excitatory synaptic input dominates the conductance change underlying the GSP. These results are in sharp contrast with the commonly accepted concept that the ongoing activity of cortical neurons reflects a shift of balance between the inhibitory and the excitatory inputs. Indeed, a concurrent increase in both type of inputs (excitation and inhibition) was previously observed (Destexhe et al., 2003; Haider et al., 2006; Rudolph et al., 2007; Okun and Lampl, 2008). On the other hand, in some reports a relatively depolarized reversal potential has been documented (Waters and Helmchen, 2006).

### THE UNIMODAL DISTRIBUTION OF THE MEASURED PARAMETERS

**Figure 5** summarizes the distribution of six parameters that were measured from our large population of neurons. The resting and reversal potentials and amplitude of the GSP are shown in the top three panels. The frequency, *R*_in_, the conductance change during the GSP are shown in the lower panel. All these parameters have unimodal distributions. The absence of multimodality shows that our large population of neurons cannot be subcategorized to specific subpopulations (see discussion).

**FIGURE 6:**
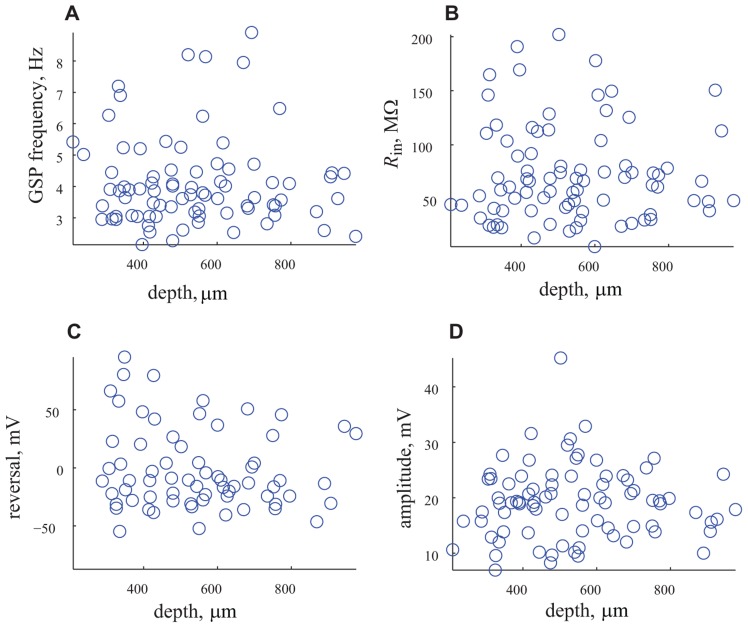
**The depth of recording does not affect the measured parameters.** **(A)** The frequency of appearance of GSP vs. depth of recording. **(B)** *R*_in_ vs. the depth of recording. **(C)** Reversal potential vs. depth of recording. **(D)** Amplitude of GSP vs. depth of recording. Note that none of the parameters are correlated with the depth of recording.

This surprising finding that challenges our long standing common agreement that the cortical layers represent different types of neurons each with its own electrical properties and connectivity needs further support. To that end we use two approaches. First we examine possible correlations between the different parameters and the depth of the recording. As shown in **Figure 6** we searched for correlations between the frequency, input resistance, reversal potential and amplitude of GSP and the depth of recording. Whereas we did not expect correlation between the frequency of the GSP and the depth of the recording, since it is a general phenomenon that encompasses the entire cortex (6A), we did expect to find correlation with the input resistance, since the size of the neurons differ in different layers. As shown in **Figure 6B** no correlation in input resistance was found. It was difficult to predict what will happen with the reversal potential and amplitude of the GSPs, but again no correlation with depth of recording was found. These results strongly support the unimodal distribution of these parameters.

**FIGURE 7:**
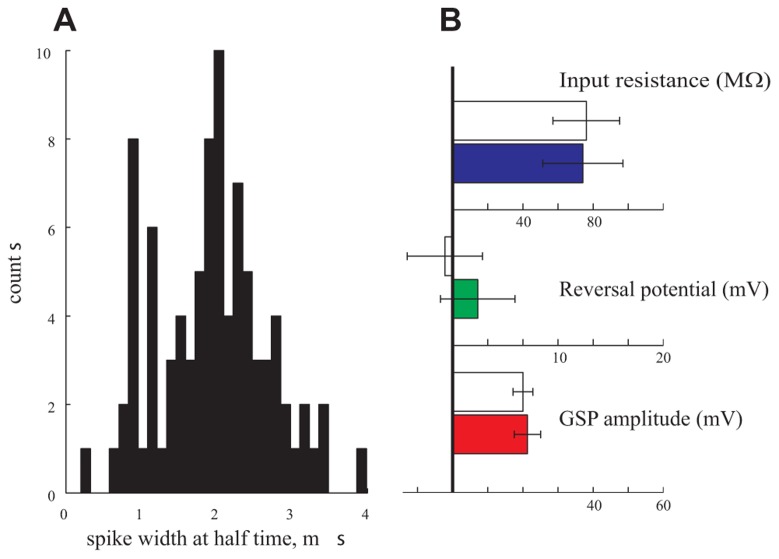
**Pyramidal neurons vs. interneurons.** **(A)** The spike duration at half width of 95 cells is bimodal pointing to a pyramidal population (with an average of 2 ms duration) and an interneuronal population (with an average of 1 ms duration). **(B)** Amplitude (red), reversal potential (green), and *R*_in_ (blue) of the two populations. Filled bars represent the pyramidal population and empty bars represent the interneuronal population.

Finally, in a further attempt to find correlations between certain parameters and the type of neurons, we classified the neurons into excitatory and inhibitory neurons. To that end we preformed a thorough analysis of the spike properties of the neurons (Nowak et al., 2003). We analyzed the distribution of the spike width of 95 cells. Indeed, as demonstrated in **Figure 7A**, a bimodal distribution was found. We assume that the cells with spike duration of ~1 ms reflect inhibitory interneurons whereas those with 2 ms spike duration represent pyramidal cells (Nowak et al., 2003). We then selected 10 cells from each group and compared their respective GSP amplitude, *R*_in_ and reversal potential. As shown in **Figure 7B** these three parameters are identical in the two groups. We conclude that, indeed, in our hands the subthreshold activity and some neuronal properties of anesthetized cortical neurons are distributed in a unimodal manner.

The reversal potential of the ongoing activity is rather high, close to zero. There are two possible explanations for such a high reversal potential. First, the ongoing activity in cortical neurons is mainly due to the contribution of excitatory inputs during the upstroke of the GSP. This explanation is in contrast with previous observations that both excitation and inhibition contribute in a balanced manner during the ongoing subthreshold activity of cortical neurons (Okun and Lampl, 2008) or that inhibitory activity is dominant in shaping the membrane potential dynamics (Rudolph et al., 2007). An alternative explanation is that our preparation in our experimental condition had a rather low inhibitory activity. Therefore, we examined the contribution of inhibition to the neurons’ activity by first, changing the chloride’s reversal potential (Sachdev et al., 2004) and second by blocking GABAergic receptors.

### SHIFTING THE INHIBITORY REVERSAL POTENTIAL WITH INTRACELLULAR CHLORIDE

The high level of the reversal potential of the GSP suggests a small contribution of inhibitory synapses. To confirm this observation, we elevated the intracellular chloride concentration to amplify the inhibitory potential. To that end the intracellular solution was modified by replacing 50% of the K^+^ in the solution with Cl^-^ in *n* = 9 cells. Chloride loading resulted in a depolarized resting potential. The average resting potential in nine cells recorded with the modified intracellular solution was -48.9 ± 23.5 mV, significantly higher than that of the cells that were recorded using a standard intracellular solution (*p* << 0.05). Surprisingly, all the other characteristics of the cell were not altered as a result of the chloride loading. The *R*_in_ (and consequently *R*_in_ change), firing frequency, GSP frequency, GSP rise time and GSP duration were not significantly different from those of the control cells (**Table 1**). The amplitude of GSPs (29.3 ± 39.7 mV, *p* = 0.023) of the chloride loaded cells was found to be higher than the amplitude of the rest of the cells; however, excluding one cell with a remarkably high resting potential, resulted in an average amplitude of 16.2 ± 6.7 mV, which is not different from that of the non-treated cells.

We conclude that apart from the resting potential, all of the parameters that were tested for the chloride loaded cells did not differ from those of the cells which were recorded from using the standard intracellular solution. This further strengthens our conclusion that the GSP reversal potential is minimally affected by inhibition.

### BLOCKING OF GABA_A_ RECEPTOR INDUCED BURSTING ACTIVITY IN CORTICAL NEURONS

**FIGURE 8:**
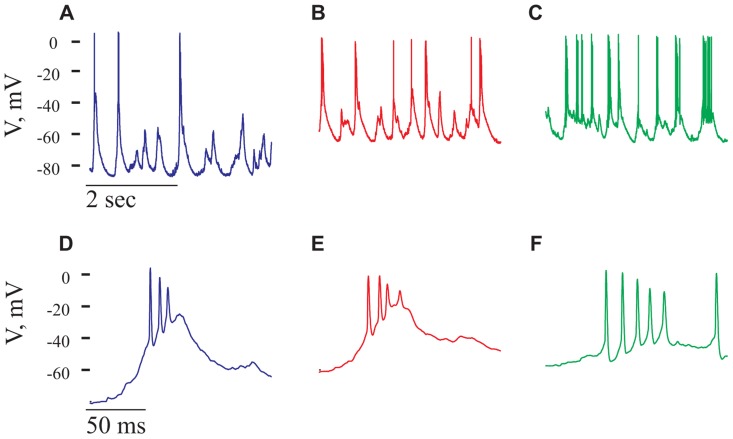
**Paroxysmal depolarizing shift, PDS, recorded in the presence of Gabazine.** All panels refer to data from the same cell after administration of gabazine (2.5 mM). **(A–C)** voltage traces recorded at three holding potentials from a neuron after gabazine administration. Note the increase in number of PDSs with depolarization. **(D–F)** Magnifications of PDSs from the corres-ponding traces in **(A–C)**. Note that the PDSs’ waveform is voltage-dependent.

Gabazine at a concentration of 2.5 μM was administered onto the surface of the cortex. Shortly thereafter (usually in less than a minute from the time of gabazine application) an increase in the spontaneous spiking activity of the cell was evident. In the cell shown in **Figure 8** this activity increased by 2.8-fold (from 0.65 to 1.8 Hz). A closer inspection of the spontaneous activity revealed that most APs were composed of a high frequency burst, resembling a paroxysmal depolarizing shift (PDS; Johnston and Brown, 1981; Bazhenov et al., 2004; Timofeev et al., 2004). PDSs were significantly larger in amplitude than the GSPs (**Figures 8A,D**) and appeared at a frequency of 1.5 ± 0.9 Hz. The PDS was characterized by a typical burst, composed of two to seven APs of variable amplitudes, with a mean inter spike interval (ISI) of 9.8 ± 2.5 ms. The increase in firing and the appearance of PDSs demonstrate that gabazine administration indeed blocked GABA_A_ receptors. Blocking of the inhibitory activity has an impact on the dynamics of the network and results in PDSs. We therefore expected to find little or no effect of the cells’ intrinsic properties on the PDSs. However, we were surprised to see that the frequency of the PDSs were governed by the cell’s resting potential. **Figures 8A–C**, demonstrates that depolarization of the cell increased the frequency of PDSs. On average, depolarizing the cells from -71 ± 8 to -48 ± 8 mV (*n* = 18) increased the frequency of PDSs by 2.8 ± 1.9-fold. As demonstrated in **Figures 8D–F**, depolarization also increased the number of APs per burst. For example, in the cell shown in **Figure 8**, the number of spikes/burst increased from 2.2 to 3.5 spikes/burst. These results clearly show that the PDSs are of intrinsic origin and therefore their frequency increases with depolarization as smaller synaptic events are capable of initiating this response. These results are in contrast with the results of Johnston and Brown (1981) that claim that PDSs result from a giant excitatory postsynaptic potential. Our results are in agreement with those of (Bazhenov et al., 2004; Timofeev et al., 2004) that show that intrinsic cellular factors have a significant role in shaping the PDSs. Clearly, blocking of inhibition has a significant effect on the ongoing activity of cortical neurons.

### THE REVERSAL POTENTIAL OF THE EVOKED RESPONSE

We used an extracellular stimulating electrode, placed within the craniotomy, to evoke voltage response in the intracellularly recorded cells. The stimulus always evoked a larger response at the resting potential than at the peak of the GSP. A similar observation was reported by Sachdev et al. (2004) to whisker stimulation. We therefore averaged only the responses at the resting potential. The average reversal potential of the evoked response was -5 ± 20.6 mV (*n* = 7). In these same cells, the average reversal potential of the GSPs was 9.6 ± 14.4 mV, not different from the reversal potential of the evoked response and not different from the GSPs’ reversal potential of the whole population of cells (*n* = 95). We then measured the reversal potential of the evoked response of chloride loaded cells, *n* = 4. The reversal potential for these cells was 19.5 ± 16.7 mV and did not differ from the reversal potential of the GSPs in these same cells (*n* = 4) and from other chloride loaded cells (*n* = 7). However, the reversal potential of the evoked response calculated for the chloride loaded cells (*n* = 4) was significantly higher than that calculated for the cells that were recorded with the standard intracellular solution (*p* = 0.04). We therefore conclude that in the evoked response, the participation of inhibitory inputs is much larger than its participation in the ongoing activity.

## DISCUSSION

### THE UNIFORM DISTRIBUTION OF THE ELECTROPHYSIOLOGICAL PROPERTIES OF CORTICAL CELLS *IN VIVO*

We recorded using whole cell patch clamp from a large population (*n* = 95) of cortical pyramidal neurons *in vivo*. All of the cells showed large, spontaneous voltage fluctuations, termed GSPs. For each cell, we characterized the basic electrophysiological properties, i.e., resting potential, spike rate and input resistance (**Figures 4 and 5**; **Table 1**). All of the parameters are distributed unimodally. The unimodal distribution is rather unexpected as it has been shown that cortical neurons can be categorized into a minimum of four physiological classes (Connors and Gutnick, 1990; Gray and McCormick, 1996; Steriade et al., 1998; Nelson et al., 2006; Ascoli et al., 2008) and six anatomical layers. As shown in **Table 1** and **Figure 6**, we have recorded from various depths and consequently from different layers. The unimodal distribution of the tested physiological parameters (see **Figure 5**) points to a non-discrete distribution of the cells’ physiological properties. Every attempt that we have made to classify the sampled neurons (*n* = 95) into subgroups with respect to the recording depth have failed (**Figure 6**). This surprising result may arise from averaging out the differences by using animals of different ages. We therefore selected a subset of the neurons, all from animals within the range of 30–40 g. These cells were distributed throughout the depth of the cortex. However, even within this subpopulation we see a unimodal distribution of the neurons’ electrical characteristics (data not shown). Hence, the wide range of animal age/weight in our hands did not account for masking the different cell classes. Also, seeking for correlations between different electrical characteristics of the cells did not yield significant correlations; none of the correlation coefficients that we had calculated exceeded the value of *r*^2^ = 0.1. It is tempting to speculate that the intrinsic properties of neurons embedded in an active network are masked by the inter-network connectivity. In this regard we should mention the work of Berg et al. (2007) demonstrating that during scratch reflex in turtles, the intrinsic properties of the cells are severely obstructed by the high conductance state resulting from the massive synaptic input.

### THE GSPs ARE MANIFESTED UNIFORMLY ACROSS THE CORTICAL LAYERS

Similar to the neurons’ physiological characteristics, the characteristics of the GSPs, i.e., reversal potential, amplitude, frequency, rise time, duration, *R*_in_ at the peak of the GSP and *R*_in_ change between the depolarized and hyperpolarized fragments of the GSP, were distributed unimodally throughout the different cortical layers (**Figure 5** and **Table 1**). We found no correlation within the characteristics of the GSPs and between the GSPs and the resting potential and input resistance of the cells. To rule out an effect of the resting potential on the amplitude of the GSP, we normalized its amplitude by the resting membrane potential (see Materials and Methods). The normalized amplitude was also found to be independent of the neurons’ depth and animals’ age. Therefore, we claim that despite the significant differences between cortical neurons, the manifestation of the GSP is uniform.

### THE ONGOING ACTIVITY IN THE ANAESTHETIZED BRAIN INVOLVES A CONDUCTANCE CHANGE

We examined whether the spontaneous rhythmic ongoing activity in the anaesthetized brain involves a change in membrane conductance. In accordance with some (Waters and Helmchen, 2006) and in contradiction with others (Pare et al., 1998; Steriade et al., 2001; Destexhe et al., 2003; Petersen et al., 2003; Crochet et al., 2005; Leger et al., 2005) we found that the changes in conductance ranges between a decrease of 35% to an increase of 76%, with an average conductance change of 22 ± 21%. We propose that GSPs are composed of individual synaptic potentials that appear at different degrees of synchrony.

### THE DEPOLARIZED REVERSAL POTENTIAL OF THE GSPs POINT TOWARD A SMALL CONTRIBUTION OF INHIBITION TO THE ONGOING ACTIVITY

Following our examination of the conductance change during GSPs, we studied its reversal potential. The reversal potential is an informative measure regarding the balance between inhibition and excitation in the active network. If, for instance, excitation was dominant in the ongoing activity, one should expect a reversal potential as measured at the soma to be several millivolts below zero (taking into account an excitation reversal potential of 0 mV and the fact that most synaptic activity occurs at distal dendrites). In contrast, if inhibition was dominant in the ongoing activity, a reversal potential closer to -70 mV is expected (Rudolph et al., 2007; Okun and Lampl, 2008). We found that the reversal potential during GSPs was -8 mV, suggesting the dominance of the excitatory processes, with a small contribution of inhibition. Given the measured -8 mV reversal potential, the maximal contribution of the inhibition is 11%, assuming that (a) both conductance changes occur at the same location; (b) the inhibitory and excitatory reversal potentials are -70 and 0 mV, respectively, and (c) the conductance changes occur in the vicinity of the recording site. This result was further supported by shifting the reversal potential of inhibition in the depolarizing direction, by replacing 50% of the K^+^ in the intracellular solution with Cl^-^. This manipulation did not shift the GSP reversal potential, further supporting the minimal contribution of chloride. Furthermore it did not alter any of the other parameters of the ongoing activity.

### INHIBITION IS PRESENT AND ACTIVE IN THE EVOKED RESPONSE DESPITE ITS ABSENCE IN THE ONGOING ACTIVITY

To directly examine the contribution of inhibition in shaping the cortical dynamics, we blocked local GABA_A_ conductance by applying gabazine onto the cortex. This dramatically changed the spontaneous activity; spiking frequency was increased and bursting activity was evident in all tested cells. Appearance of bursts of spikes, resembling PDSs, was observed shortly after gabazine administration. Because the frequency of the bursts increases with depolarization we suggest that they are governed by an intrinsic property of the cells. The powerful effect of blocking of inhibition on the cortical activity clearly demonstrated the potency of the inhibition in these circuits. Therefore, we sought for inhibitory activity in the evoked response. We used an extracellular stimulating electrode to evoke a voltage response. The stimulus always evoked a larger response at the resting potential than at the peak of the GSP, similar to the response obtained by Sachdev et al. (2004) and Wilson and Kawaguchi (1996) to whisker stimulation. The average reversal potential of the evoked response was -5 mV, similar to the reversal potential of the GSPs in the same cells as well as in the whole population of cells. However, the reversal potential of the evoked response of chloride loaded cells was 19.5 mV, not different from the reversal potential of the GSPs in these same cells and from other chloride loaded cells, but significantly higher than the evoked response of the cells that were recorded with the standard intracellular solution (*p* = 0.04). Therefore, in the evoked response, in contrast to the ongoing activity, inhibition plays a conspicuous role.

### POSSIBLE USES FOR THE ABSENCE OF INHIBITION IN THE ONGOING ACTIVITY

The known physiological and/or the anatomical subclasses of cells that are known to exist in the cortex were not exposed by the electrical measures during ongoing activity. This strengthens the assumption that the four (or more) main physiologically categorized neocortical neuronal classes are functionally flexible. The mere definition of an electrical class of cells may depend on the state of the animal. As has been proposed previously by Steriade (2003),^2004^, we assume that firing patterns can be transformed from one type into another by slight changes in membrane potential. Furthermore, the difference between electrical activities of different neuronal types is much reduced during periods of wakefulness. We do not dispute hereby the anatomical differences between cells within and between cortical layers; we therefore conclude that the GSPs are a phenomena generated by the network that camouflages the intrinsic electrical properties of the cells as was previously suggested by Berg et al. (2007). This is in agreement with Sakata and Harris (2009) who claim that it is unclear how anatomical differences affect the laminar structure of spontaneous and evoked population activity.

The ongoing activity in the anaesthetized brain represents an important mode of cortical activity. This activity has been shown to be similar to the activity during slow wave sleep, in which the cortex dwells for 25–50% of the 24-h cyclic period (more during development and less in later stages in life). The absence of a strong inhibitory impact in the ongoing activity during sleep or anesthesia may drive the system to a non-equilibrium state, allowing the system to reach stable attractors which otherwise would have been out of its scope. Hypothetically, this can enable the formation of original functional connectivity which otherwise would have been inhibited. Whilst the evoked response is almost completely inhibited in the depolarized phase of the ongoing activity, spontaneous activity is free to occur at all phases, as inhibition is absent. This may permit plasticity in non-trivial paths. It is yet to be determined whether the ongoing activity in awake animals is similar to that of anaesthetized ones or the lack of inhibition is a privilege of the anaesthetized state.

## Conflict of Interest Statement

The authors declare that the research was conducted in the absence of any commercial or financial relationships that could be construed as a potential conflict of interest.
